# Interferon-alpha therapy for refractory kaposiform hemangioendothelioma: a single-center experience

**DOI:** 10.1038/srep36261

**Published:** 2016-10-31

**Authors:** Hai Wei Wu, Xuan Wang, Ling Zhang, Hai Guang Zhao, Yan An Wang, Li Xin Su, Xin Dong Fan, Jia Wei Zheng

**Affiliations:** 1 Department of Oral and Maxillofacial Surgery, Shanghai Ninth People’s Hospital, College of Stomatology, Shanghai Jiao Tong University School of Medicine, Shanghai 200011, China; 2 Department of General Dentistry, Shanghai Ninth People’s Hospital, College of Stomatology, Shanghai Jiao Tong University School of Medicine, Shanghai 200011, China; 3 Department of Vascular Surgery, Shanghai Ninth People’s Hospital, Shanghai Jiao Tong University School of Medicine, Shanghai 200011, China; 4 Department of Interventional Radiotherapy, Shanghai Ninth People’s Hospital, Shanghai Jiao Tong University School of Medicine, Shanghai 200011, China

## Abstract

Kaposiform hemangioendothelioma (KHE) is a relatively rare vascular tumor with an aggressive and infiltrating nature. Previous studies have revealed an exclusive relationship between KHE and Kasabach-Merritt Phenomenon (KMP), which is associated with high morbidity and mortality. No universally accepted treatment modality exists for refractory KHE with or without KMP. The aim of this study was to evaluate the safety and efficacy of interferon-alpha (IFN-α) therapy for treatment of refractory KHE. Twelve consecutive patients with KHE were treated with subcutaneous injections of IFN-α after other treatments had failed. Eleven patients exhibited a reduction in tumor size of more than 50%, and the platelet count for all five patients with KMP returned to normal level after IFN-α therapy. The duration of IFN-α treatment ranged from 3 months to 9 months (mean: 6.3 months). The response time for IFN-α treatment ranged from 10 days to 5 weeks (mean: 3.6 weeks). Additionally, no severe complications, such as neurological damage or spastic diplegia, were observed in these patients. In conclusion, our study suggested that IFN-α therapy is effective and safe for refractory KHE, and IFN-α may be used as an alternative after other treatments have failed.

Kaposiform hemangioendothelioma (KHE) is a rare, locally aggressive vascular tumor that typically affects infants. KHE is usually associated with cutaneous lesions in the extremities, torso and cervicofacial region^1^. Occasionally, some lesions could infiltrate subcutaneous tissue, including the bone, mediastinum and retroperitoneum^2^. Theses lesions are characterized by rapid growth and an infiltrating nature that may potentially lead to high morbidity and mortality. Clinically, KHE often appears as erythematous-violaceous masses or plaques with ill-defined margins. According to a retrospective review of 107 patients, the typical clinical features of KHE includes an enlarging mass, thrombocytopenia, and pain or functional disturbances^3^. Histologically, KHE is composed of solid nodules that are a mixture of spindle-shaped endothelial cells and small capillary vessels. The typical magnetic resonance imaging (MRI) presentation of KHE is homogeneous hyperintense in T2-weighted sequences and isointense in T1-weighted sequences^4^. Numerous studies have revealed an exclusive relationship between KHE and Kasabach-Merritt Phenomenon (KMP), which is characterized by consumptive coagulopathy and thrombocytopenia with enlarging vascular tumors, including KHE and tufted angioma (TA)^5^. Hemorrhage, disturbance of homeostasis and uncontrollable growth of vascular lesions usually lead to poor therapeutic outcomes in patients with KMP^6^. Using a clinical-laboratory analysis, Croteau *et al*. found that more than 70% of KHE patients develop KMP eventually. KHE that infiltrates into deeper anatomic regions is more likely to manifest KMP^3^. The molecular mechanism underlying this phenomenon has not been well established, but it is presumed that endothelial cells in KHE have a unique ability to trap platelets and then stimulate the release of angiogenic factors sequestered by platelets^5^.

Given the relative rarity of KHE, no universally accepted treatment modality currently exists. A diverse range of treatments have been applied in the treatment of KHE, including surgery, arterial embolization, physical compression, laser, radiotherapy and medical therapy^5^. Moreover, individual responses to various treatments differ considerably. Interferon-alpha (IFN-α) has been used in the treatment of complicated vascular tumors for several decades. Our previous study has reported the successful treatment of alarming hemangioma with IFN-α^7^. However, the use of IFN-α in KHE treatment has been controversial because of its potential side effects in infants^8^,^9^,^10^. In this study, we sought to evaluate the efficacy and safety of IFN-α for the treatment of refractory KHE in a series of 12 consecutive patients.

## Materials and Methods

### Patients

The study population consisted of 12 consecutive patients with KHE who received IFN-α treatment between July 2008 and June 2015 at the Department of Oral and Maxillofacial Surgery, Shanghai Ninth People’s Hospital, College of Stomatology, Shanghai Jiao Tong University School of Medicine. Our study was approved by the Institute Review Board of Shanghai Ninth People’s Hospital and conducted in accordance with approved guidelines. Informed consent was obtained from all parents of the patients. The diagnosis of KHE with or without KMP was confirmed on the basis of clinical features, characteristic imaging results, laboratory data and tissue biopsy results. A thorough history was obtained from each patient regarding their previous treatment course.

### Dosage

All patients were treated with a subcutaneous injection of IFN-α, administered once per day. The initial dosage was set at 1 × 10^6^U/m^2^/day for the first week. Then, IFN-α was administered at a dosage of 3 × 10^6^U/m^2^/day for a period of 3–9 months. The objective of the treatment was to control tumor growth and reestablish platelet homeostasis. Periodic blood tests and neurological examinations were performed during the treatment course. The therapy was gradually tapered off by increasing the interval and halving the dosage.

### Outcome measurement

No standard methods exist for outcome measurement of KHE. In this study, the therapeutic efficacy of treatment of KHE without KMP was evaluated primarily on the basis of the percentage of lesion regression, and for KHE with KMP, the therapeutic efficacy was assessed primarily on the basis of platelet counts and lesion regression. Assessment of the percentage of lesion regression was based on clinical photographs and imaging results (including Doppler ultrasonography and MRI). Two other independent physicians completed the evaluation. The platelet count is crucial in evaluating the therapeutic efficacy of KHE with KMP. Normalization of platelet count after treatment was set at 100 × 10^9^/L. All patients were followed up, and the follow-up period ranged from 6 months to 2 years.

## Results

### Clinical and histological features

**Figure 1:**
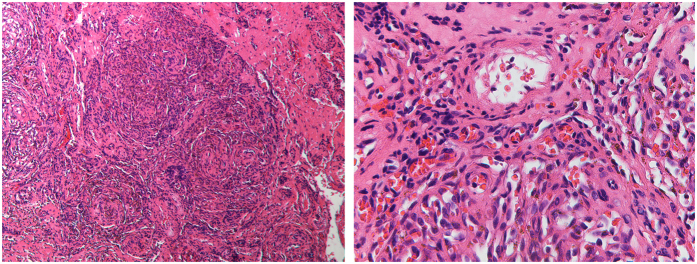
Typical histological photomicrographs of KHE. The low power view shows nodules of spindle-shaped endothelial cells resembling Kaposi sarcoma. The higher magnification shows tightly packed spindle-shaped cells and small capillaries.

Clinical data of the patients are listed in Table 1. Seven male and 5 female patients were included in the study. The diagnosis was confirmed for all patients by histological examination prior to treatment. The mean age of KHE onset was 2.4 months with a range from 20 days to 8 months. The anatomical sites of these lesions included 8 in the cervicofacial region, 2 in the upper limb, 1 in the lower limb and 1 on the back. No lesions involved multiple anatomical locations. Eleven of 12 patients had cutaneous vascular lesions that manifested as indurated purple masses, and only one patient lacked local cutaneous swelling. The typical histological features of KHE are shown in Fig. 1, in which the tumor is dominated by nodules of spindle-shaped endothelial cells resembling Kaposi sarcoma, and slit-like capillaries can be seen between tightly packed spindle-shaped endothelial cells.

### Previous treatments

Patients in this series had received several treatment modalities prior to IFN-α treatment (shown in Table 1). Those treatments included surgery for 1 patient, corticosteroids for 2 patients, propranolol for 1 patient, corticosteroids combined with propranolol for 4 patients, and corticosteroids combined with vincristine for 4 patients. No apparent therapeutic responses were achieved with these therapies.

### Therapeutic outcomes

**Figure 2:**
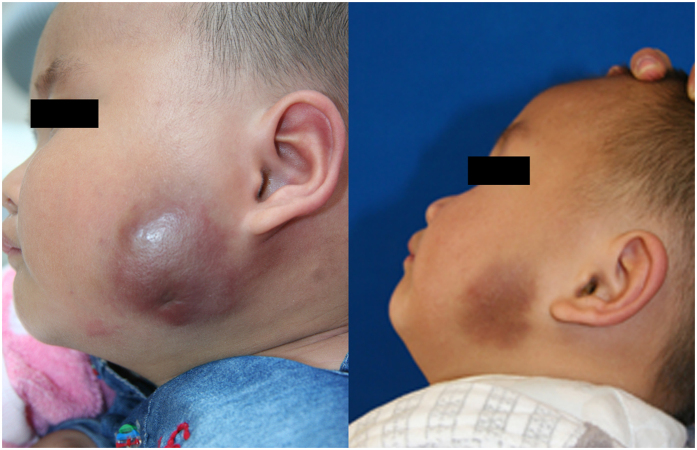
Response of refractory KHE without KMP to IFN-α therapy in patient No. 1. (**A**) Before the onset of IFN-α therapy at an age of 6 months; (**B**) Three months after the end of IFN-α therapy at an age of 18 months. Obvious discoloration and regression in tumor size were observed.

**Figure 3:**
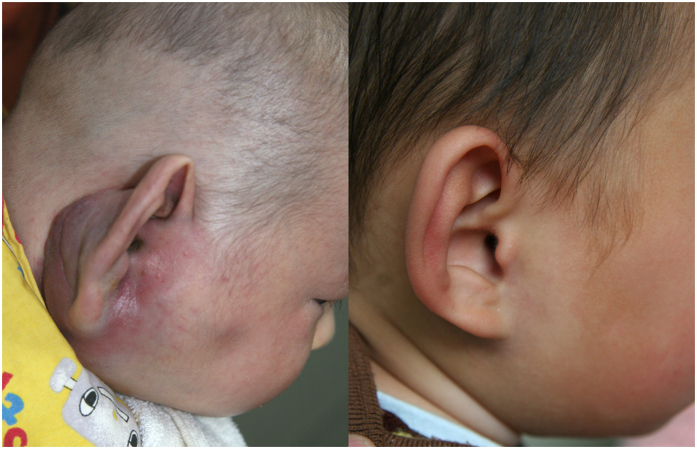
Response of refractory KHE with KMP to IFN-α therapy in patient No. 8. (**A**) Before the onset of IFN-α therapy at an age of 3 months; (**B**) Twenty months after the end of IFN-α therapy at an age of 29 months. Complete regression in the size and symmetric appearance was obtained without recurrence.

Table 2 outlines the therapeutic outcomes of 12 patients who received IFN-α treatment. The duration of IFN-α treatment ranged from 3 to 9 months (mean: 6.3 months). The response time to IFN-α treatment ranged from 10 days to 5 weeks (mean: 3.6 weeks). All five patients with KMP achieved normalization and stabilization of the platelet count after treatment. Significant regression in the size and color of vascular lesions was observed 11 patients. Overall, 9 patients (75.0%) had excellent responses, with more than 80% regression of KHE lesions, and 2 patients (16.7%) had good responses, with more than 50% regression of KHE lesions (typical therapeutic responses are shown in Figs 2 and 3). One KHE lesion did not respond to IFN-α treatment, and we therefore applied sirolimus treatment for this patient with refractory KHE. Seventy percent regression of the lesion was achieved. The mean follow-up time was 13.2 months (ranging from 6 months to 2 years). At the most recent follow-up, no patients required additional IFN-α or another treatment, and no recurrences of the lesion or the coagulopathy were observed.

### Complications

No severe adverse effects were observed during IFN-α treatment. The main complications included mild fever in 5 patients (41.7%), diarrhea in 1 patient (8.3%), and anorexia in 1 patient (8.3%). No patients experienced neurological damage, especially spastic diplegia.

## Discussion

Unlike common infantile hemangiomas, spontaneous involution seldom occurs in patients with KHE. KMP, as first reported by Kasabach and Merritt in 1940, refers to vascular lesions with complications of thrombocytopenia and coagulopathy. Moreover, the majority of vascular lesions with KMP have been shown to be more aggressive KHE^11^. Uncontrolled enlarging KHE and KHE with KMP typically require active intervention. Early surgical intervention has been shown to be effective and has diagnostic value for the treatment of small KHE tumors^12^. However, surgery is often not feasible for most KHEs, owing to infiltration of deep tissues and severe complications, such as hemorrhage, secondary deformity and possible nerve damage. Currently, surgery is often used as a supplement to medical therapies for the treatment of refractory KHE^13^. Arterial embolization can achieve an immediate therapeutic effect for life-threatening KHEs with KMP through embolization of the main feeding artery, and it is usually an important part of stepwise multimodal therapy that improves the efficacy of the medical therapy^14^. Notably, the current treatment for KHE is primarily a comprehensive treatment pattern based on medical therapies.

Many medical therapies have been attempted for the treatment of KHE, including administration of corticosteroids, vincristine, propranolol, aspirin, sirolimus and IFN-α^5^. Systemic corticosteroids have been widely used as a first-line therapy for the treatment of KHE with or without KMP. However, individual responses to systemic corticosteroids range from no effect to obvious regression of the lesions^15^,^16^. Several studies have demonstrated significant response of steroid-resistant KHE with KMP to vincristine^17^,^18^. Furthermore, a consensus-derived therapy standard suggests that oral prednisolone and intravenous vincristine are an optimal choice for KHE treatment^2^. However, the therapeutic effects in 4 patients in this series who had received corticosteroids combined with vincristine were not apparent. Propranolol, a non-selective β-adrenergic receptor blocker, has been successfully used in the treatment of infantile hemangioma. Recently, Hermans *et al*. have reported a novel use of propranolol for the management of KHE with KMP^19^. Further clinical investigation has revealed that the therapeutic effects of propranolol vary, and in a study by Chiu *et al*., less than 40% of patients had obvious responses to propranolol treatment^20^.

In this study, subcutaneous injections of IFN-α were applied as a treatment after failure of other therapies. IFN-α has previously been used as an antiviral agent to regulate immune responses through cytokines. Several studies have shown that IFN-α inhibits tumor angiogenesis by down-regulating the secretion of angiogenic molecules, such as vascular endothelial growth factor (VEGF), fibroblast growth factor (FGF) and platelet-dependent growth factor (PDGF)^21^,^22^. White *et al*. firstly reported successful management of pulmonary hemangiomas with IFN-α in 1989^23^. Since then, IFN-α has been increasingly used for the treatment of vascular tumors. In our previous report of using IFN-α for alarming infantile hemangiomas^7^, all patients exhibited a clear reduction in tumor size, and no severe adverse effects were observed. Several studies have reported the use of IFN-α therapy as a treatment for KHE, but with variable therapeutic outcomes^24^,^25^. In the present study, we investigated the results of IFN-α therapy for refractory KHE in 12 consecutive patients who had experienced failure of various treatments prior to IFN-α therapy. After IFN-α administration, significant regression of vascular lesions was achieved in 11 patients (91.7%), and all 5 patients with KMP obtained substantial resolution of their thrombocytopenia. Only one patient responsed poorly to IFN-α therapy. Recently, many studies have shown attractive and promising effects of mechanistic target of rapamycin (mTOR) inhibitors in the treatment of refractory KHE^26^,^27^. We reserved sirolimus as an off-table regime after failure of IFN-α therapy, and achieved 70% regression of the refractory lesion without relapse within 6 months. Compared with IFN-α therapy for alarming infantile hemangiomas, which has a mean duration of 3 months^7^, the duration of IFN-α therapy for refractory KHEs was longer, indicating that KHE is more difficult to manage than infantile hemangiomas. The safety of IFN-α for the treatment of KHEs has been attracting the attention of pediatricians because of the potentially severe complications reported by several investigators^10^,^28^. In this series, the main complications were mild fever in 5 patients (41.7%), which was resolved within 3–5 days without specific intervention. Furthermore, no neurological damage especially spastic diplegia were found in our study after a follow-up of at least 6 months. This result is probably because the duration of IFN-α therapy in our study was limited to 36 weeks, whereas the maximum durations of IFN-α therapy in previous studies that have reported severe complications induced by IFN-α have been as long as 103 weeks^28^ and 120 weeks^10^. Nevertheless, further long-term clinical observation of the risk factors for potential neurological damage during IFN-α therapy is needed in the future.

In conclusion, this study presents our experience of applying IFN-α therapy as a treatment for refractory KHEs, impressive responses with mild complications after IFN-α therapy were observed in this study. The results suggest that IFN-α therapy may be an effective and safe means for treating refractory KHEs.

## Additional Information

**How to cite this article**: Wu, H. W. *et al*. Interferon-alpha therapy for refractory kaposiform hemangioendothelioma: a single-center experience. *Sci. Rep*. **6**, 36261; doi: 10.1038/srep36261 (2016).

**Publisher’s note:** Springer Nature remains neutral with regard to jurisdictional claims in published maps and institutional affiliations.

## Figures and Tables

**Table 1:** Clinical feature and previous treatment of 12 patients with kaposiform hemangioendothelioma.

Patient No. And Condition	Sex	Age of Onset (months)	Location of Lesion	Previous Treatment
KHE Without KMP				
1	F	2	Left parotid region	Corticoteroids, Propranolol
2	M	8	Left maxilla	Surgery
3	M	2	Left parotid region	Corticoteroids, Propranolol
4	F	1	Right upper limb	Corticoteroids
5	M	3	Left cheek	Propranolol
6	M	3	Right neck	Corticoteroids, Propranolol
7	F	2	Left submandibular region	Corticoteroids, Vincristine
KHE With KMP				
8	F	2	Right parotid region	Corticoteroids
9	M	3	Left lower limb	Corticoteroids, Vincristine
10	F	1	Left parotid region	Corticoteroids, Vincristine
11	M	1	Right upper limb	Corticoteroids, Vincristine
12	M	20 days	Left back	Corticoteroids, Propranolol

**Abbreviations**: KHE, Kaposiform Hmangioendothelioma; KMP, Kasabach–Merritt Phenomenon; F, female; M, male; IFN-α, Interferon alpha.

**Table 2:** Clinical outcome of 12 patients with kaposiform hemangioendothelioma.

Patient No. And Condition	Duration of IFN-α Treatment (months)	Response Time to IFN-α Treatment (weeks)	Platelet Count Before IFN-α Treatment	Platelet Count After IFN-α Treatment	Complication	Outcome of Vascular Lesion
KHE Without KMP						
1	9	3	normal	normal	None	80% regression
2	6	2.5	normal	normal	Mild fever	90% regression
3	3	—	normal	normal	Diarrhea	No responses*
4	6	3	normal	normal	Anorexia	95% regression
5	6	4	normal	normal	None	90% regression
6	9	2	normal	normal	None	80% regression
7	6	4	normal	normal	Mild fever	80% regression
KHE With KMP						
8	6	5	35 × 10^9/L	256 × 10^9/L	Mild fever	100% regression
9	9	3	48 × 10^9/L	318 × 10^9/L	None	80% regression
10	4	10 days	17 × 10^9/L	225 × 10^9/L	None	90% regression
11	6	3	24 × 10^9/L	202 × 10^9/L	Mild fever	50% regression
12	6	4	25 × 10^9/L	247 × 10^9/L	Mild fever	60% regression

**Abbreviations**: KHE, Kaposiform Hmangioendothelioma; KMP, Kasabach–Merritt Phenomenon; F, female; M, male; IFN-α, Interferon alpha.

^*^70% regression after sirolimus treatment.
